# Directed evolution generates an oncolytic Sindbis virus with enhanced tumor-killing capacity in osteosarcoma

**DOI:** 10.1016/j.omton.2025.201096

**Published:** 2025-11-19

**Authors:** Kangyixin Sun, Qinghan Wang, Li Li, Hongjun Mei, Zijun Rao, Jia Yang, Fan Jia, Fuqiang Xu

**Affiliations:** 1 Wuhan National Laboratory for Optoelectronics, Huazhong University of Science and Technology, Wuhan 430074, China; 2 Guangdong Provincial Key Laboratory of Brain Connectome and Behavior, CAS Key Laboratory of Brain Connectome and Manipulation, the Brain Cognition and Brain Disease Institute, Translational Research Center for the Nervous System, Shenzhen Institutes of Advance Technology, Chinese Academy of Sciences, Shenzhen-Hong Kong Institute of Brain Science-Shenzhen Fundamental Research Institutions, Shenzhen 518055, China; 3 NMPA Key Laboratory for Research and Evaluation of Viral Vector Technology in Cell and Gene Therapy Medicinal Products, Key Laboratory of Quality Control Technology for Virus-Based Therapeutics, Guangdong Provincial Medical Products Administration, Shenzhen Key Laboratory of Viral Vectors for Biomedicine, Shenzhen Institutes of Advance Technology, Chinese Academy of Sciences, Shenzhen 518055, China; 4 Department of Orthopaedics, The Fifth Hospital of Wuhan, Wuhan 430050, China; 5 University of Chinese Academy of Sciences, Beijing 100049, China; 6 Faculty of Life and Health Sciences, Shenzhen University of Advanced Technology, Shenzhen 518106, China

**Keywords:** MT: Regular Issue, osteosarcoma, oncolytic virus, Sindbis virus, alphavirus, directed evolution

## Abstract

Osteosarcoma, a common cancer in adolescents, is characterized for its strong propensity for metastasis, which complicates treatment and results in a poor survival rate. Despite established therapies, the challenge remains to effectively target metastatic tumors. Oncolytic viruses offer a novel therapeutic approach by selectively infecting and destroying cancer cells while triggering antitumor immune responses. Sindbis virus (SINV), although effective in various tumor types, exhibits limited efficacy in osteosarcoma due to insufficient replication within these cells. This study aimed to improve therapeutic efficacy of SINV through directed evolution. By passaging the virus in osteosarcoma cell lines (HOS and U2OS), we developed an adaptive variant, designated SINV-P438L, with a mutation in nonstructural protein 2 (nsP2) that increased viral replication and transcription efficiency. SINV-P438L exhibited improved infectivity and cytotoxicity in osteosarcoma cells, inducing significantly activated apoptosis-associated signals compared with wild-type SINV. In animal models, SINV-P438L demonstrated superior antitumor effects in osteosarcoma and other tumor models, without causing detectable damage to normal tissues. These findings support SINV-P438L as a promising candidate for osteosarcoma treatment, with potential applications across additional cancer types.

## Graphical abstract

**Figure:**
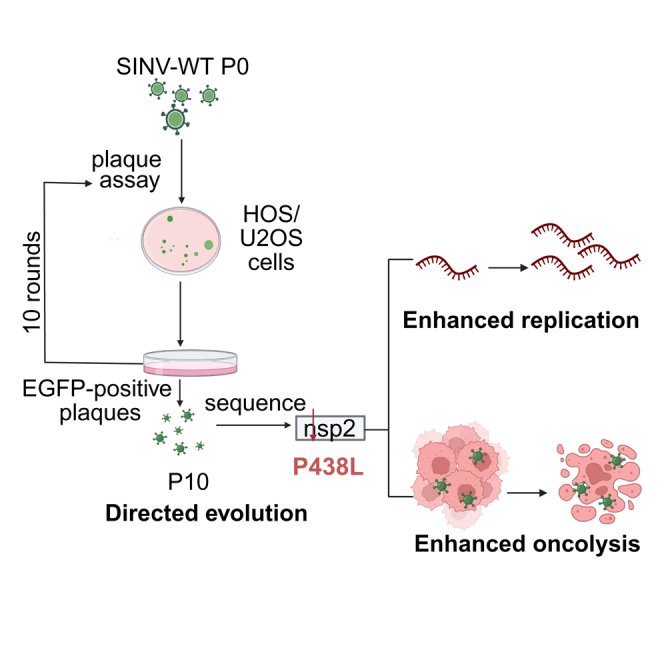

## Teaser

Directed evolution of Sindbis virus generated the P438L variant, which shows enhanced replication and selective oncolysis in osteosarcoma and hepatocellular carcinoma models. These findings highlight the potential of viral evolution strategies to tailor oncolytic viruses for precision cancer therapy.

## Introduction

Osteosarcoma is a common primary malignant tumor that most frequently occurs in adolescents and can develop in any bone throughout the body.^1^ Standard therapies based on surgery and chemotherapy have been established for osteosarcoma since the 1980s, but the high propensity of osteosarcoma cells for metastasis contributes to the low survival rate following tumor metastasis.^2^^,^^3^ Lung and bone metastases, which are prevalent in osteosarcoma, complicate the detection of micrometastases in patients, resulting in poor prognosis due to the failure of conventional therapeutic strategies to fully eliminate the tumor lesions.^1^^,^^4^^,^^5^ Therefore, researchers are actively exploring more effective treatment options. Notably, immunotherapy has shown advantages in suppressing metastasis,^6^^,^^7^ providing a valuable approach for osteosarcoma treatment.

Oncolytic viruses (OVs), a form of immunotherapy, represent a cancer treatment modality in which engineered viruses selectively replicate within tumors to induce lysis.^8^^,^^9^ These viruses can also deliver therapeutic genes and stimulate immune responses, thereby enhancing the tumor-killing efficiency.^10^^,^^11^^,^^12^ To date, four OVs, herpes simplex virus-based T-VEC, Delytact, adenovirus-based H101, and the natural coxsackievirus Rigvir, have been approved for clinical use in gliomas, melanomas, and other malignancies.^13^^,^^14^^,^^15^^,^^16^ In addition, many preclinical studies on OVs are ongoing, although few have focused on osteosarcoma.^17^^,^^18^^,^^19^^,^^20^^,^^21^ In our previous studies, we focused on the development and application of Sindbis virus (SINV) vectors in tumor therapy. Therefore, we selected SINV as the basis for establishing a SINV-based therapeutic strategy for osteosarcoma.

SINV is an enveloped, positive-strand RNA virus of the genus *Alphavirus*, encoding four nonstructural proteins (nsP1-nsP4) and five structural proteins (capsid, E3, E2, 6K, and E1).^22^^,^^23^ SINV is a blood-borne virus that spreads through the body via the bloodstream and cause fever, arthritis, and other mild symptoms after infection.^24^^,^^25^ Our previous work demonstrated that neither intracranial nor intravenous injection of SINV caused acute injury in experimental animals.^26^ The 67 kDa laminin receptor protein (LAMR), a viral receptor for SINV,^27^ is highly expressed in a variety of human tumor tissues, including breast, ovarian, lung, and colon cancers,^28^^,^^29^^,^^30^^,^^31^^,^^32^^,^^33^ contributing to the broad-spectrum antitumor activity of SINV. Moreover, SINV can activate antitumor immune responses, making it a potential candidate as an oncolytic virus.^34^^,^^35^ Several studies have explored the therapeutic potential of SINV, including significant regression of cervical and ovarian tumors following SINV treatment and prolonged survival of glioma-bearing mice treated with SINV carrying IL-12 and IL-7.^26^^,^^36^ Additionally, SINV can also induce cytopathic effects (CPEs) in a variety of tumor cells, including neuroblastoma cells and oral squamous cell carcinoma cell lines.^36^^,^^37^^,^^38^ However, the limited susceptibility of certain tumor cells to SINV restricts its oncolytic potential. Previous reports have shown that SINV replication is suppressed in osteosarcoma cells.^39^^,^^40^ Enhancing the efficiency of infection and replication of virus in these cells could not only enable viral eradication of refractory tumors but also broaden the clinical utility of SINV as an OV.

In this work, we obtained an adaptive SINV by serial passaging in osteosarcoma cell lines. We identified a critical mutation (P438L) in nsP2, which enhanced viral replication and transcription efficiency, thereby enhancing the therapeutic efficacy of SINV in solid tumors as well as metastatic cancers.

## Results

### Direction evolution improves SINV killing of osteosarcoma cell lines

**Figure 1:**
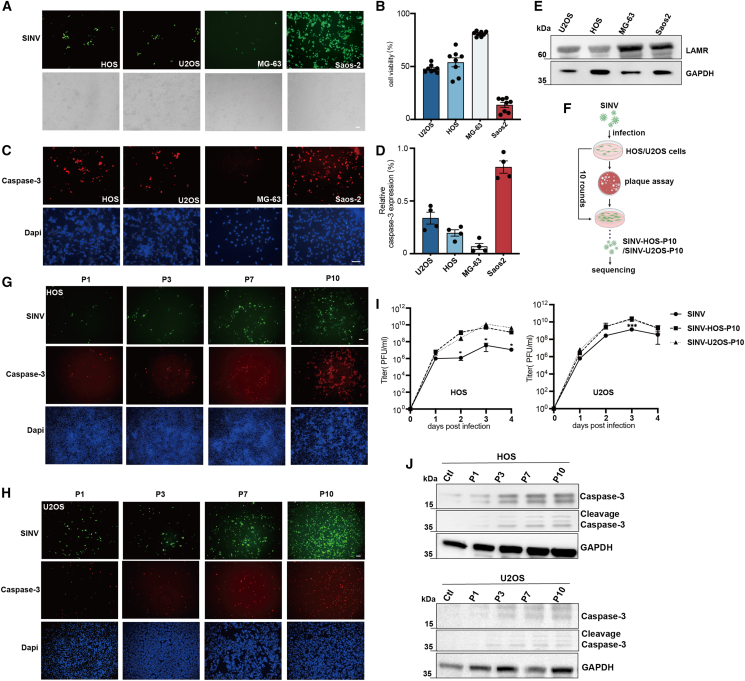
SINV passages on osteosarcoma cells to select adaptive variants (A) Osteosarcoma cells were infected with SINV (MOI = 0.1), and representative images were obtained at 24 hpi. Scale bar, 100 μm. (B) Cell viability of HOS, U2OS, MG-63, and Saos-2 cells at 48 hpi (*n* = 8). (C) Caspase-3 expression was examined in four osteosarcoma cell lines following viral infection using immunofluorescence with the Caspase-3 antibody (no. 9662, Cell Signaling Technology). Caspase-3-positive cells were stained red, while live cells were counterstained with DAPI (blue). The same antibody was used for caspase-3 detection in (G, H, and J). The percentage of caspase-positive cells relative to live cells is shown in the (D). (E) The results of western blot for four osteosarcoma cell lines expressing 67-kD LAMR. (F) Schematic diagram of SINV passaging. Each passage generation was conducted in biological triplicates to ensure reproducibility. (G and H) Representative EGFP expression and caspase-3 staining in HOS and U2OS cells infected with progeny viruses (P1, P3, P7, P10, MOI = 0.1, 48 hpi). Scale bars, 100 μm. (I) Viral titers of WT SINV, SINV-HOS-P10, and SINV-U2OS-P10 in HOS and U2OS cells (*n* = 3). ∗*p* < 0.05, ∗∗*p* < 0.01, ∗∗∗*p* < 0.001. (J) Western blot analysis of caspase-3 and cleaved caspase-3 expression in HOS and U2OS cells at 48 hpi following infection with progeny viruses.

To select suitable osteosarcoma cell lines for viral passaging, four types of osteosarcoma cells (MG-63, U2OS, HOS, and Saos-2) were infected with SINV-EGFP at 0.1 multiplicity of infection (MOI), and infection efficiency and cell viability were assessed. EGFP expression following viral infection is shown in Figure 1A. The results indicated that SINV exhibited the highest sensitivity in Saos-2 cells, while infection was minimal in MG-63. Intermediate levels of infection were observed in HOS and U2OS cells, as further confirmed by cell viability assays in Figure 1B. In addition, apoptosis-associated signals induced by viral infection was quantified by caspase staining at 48 h post-infection (hpi), the results showed that SINV efficiently induced more caspase-positive cells in Saos-2 cells, whereas the lowest infection efficiency and limited apoptosis-associated signals hindered their suitability for passaging evolution (Figures 1C and 1D). In contrast, HOS and U2OS cells, which showed moderate infection susceptibility, were identified as appropriate models for viral adaptation. Furthermore, we examined the SINV receptor on these four cells and found high expression of LAMR on the cells (Figure 1E), supporting the feasibility of serial viral passaging in HOS and USO2 cells. We subsequently conducted serial passaging of SINV in HOS and U2OS cells. In each generation, three larger plaques exhibiting strong green fluorescence were selected for propagation, as shown in Figure 1F. This was continued until after the 10th generation. Among them, representative images of P1, P3, P7, and P10 virus-infected cells were compared at 48 hpi, and demonstrated a progressive increase in viral infection efficiency with successive passages (Figure 1F). As shown in Figures 1G and 1H, one-step growth curves analyses revealed that P10 SINV displayed significantly enhanced replication in both HOS and U2OS cells compared with the wild-type (WT) virus, especially at 3 days post-injection. Moreover, the expression levels of caspase-3 protein in cells infected with the progeny virus are shown in Figure 1J. The results of caspase expression indicate that SINV infection significantly activates caspase-dependent apoptotic pathways in HOS and U2OS cells, with gradual increases in intracellular caspase-3 and cleaved caspase-3 expression observed across successive viral passages.

Based on these results, it is shown that that directed evolution successfully generated SINV variants with improved infectivity and caspase-inducing capacity in HOS and U2OS cells. Therefore, we performed whole-genome sequencing of six P10 SINVs to identify mutations underlying these phenotypic enhancements.

### The P438L mutation increases the tumor-killing efficiency of SINV on osteosarcoma

**Figure 2:**
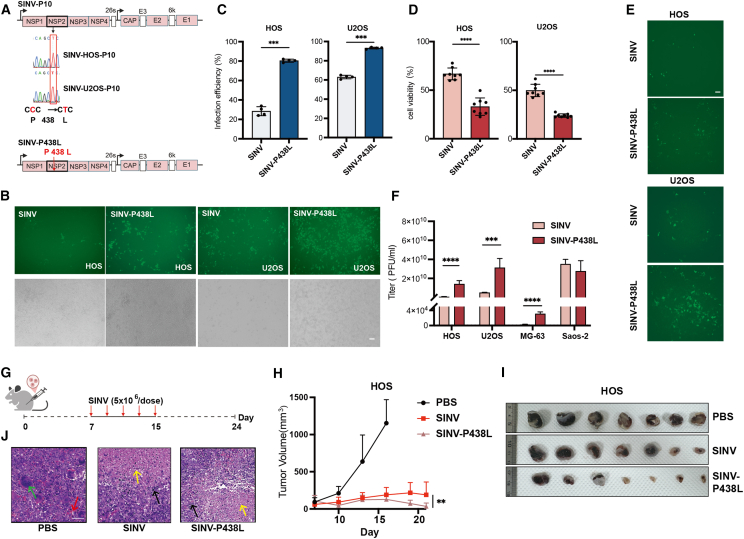
The P438L mutation on Nsp2 improves the antitumor effect of SINV in osteosarcoma (A) Mutation sites in nsP2 and schematic of SINV-P438L construction. (B) Representative images of HOS and U2OS cells infected with SINV or SINV-P438L at MOI of 0.1, 24 hpi. Scale bar, 100 μm. (C) Flow cytometry analysis of GFP positivity (*n* = 4). ∗∗∗*p* < 0.001, ∗∗∗∗*p* < 0.0001. (D) HOS and U2OS cells were infected with SINV or SINV-P438L at MOI of 0.1, and cell viability was measured 72 h later using the MTT cell proliferation and cytotoxicity assay kit (*n* = 8). (E) Plaque assay showing viral spread after infection. Scale bars, 250 μm (left) and 100 μm (right). (F) Viral titers in osteosarcoma cells at 48 hpi (*n* = 4). (G and H) Tumor xenograft growth and volumes in HOS-bearing mice (*n* = 7). (I) Resected tumor tissues at day 24. (J) H&E staining of tumor tissues. Yellow arrows indicate tumor cells, red arrows indicate infiltrating inflammatory cells, green arrows indicate hair follicle plugs, and black arrows indicate additional inflammatory cell infiltration. Scale bars, 50 μm. In SINV-treated tumor tissues, tumor cells exhibited marked necrosis with karyolysis and dissolution, accompanied by limited inflammatory infiltration. In contrast, SINV-P438L-treated tumors displayed extensive necrosis, with nuclear dissolution and only residual tumor cell outlines remaining.

After genomic analysis the P10 progeny virus, six viruses were found to share common mutation compared with the WT virus. This mutation was a C2992T substitution in the nucleotide sequence of nonstructural protein 2 (nsP2) caused the mutation from proline (Pro, P) to leucine (Leu, L) at position 438 (Figure 2A). Based on the enhanced infectivity of osteosarcoma cell lines conferred by this mutation, we hypothesized that this single substitution would improve SINV performance in tumor cell lines. Therefore, we constructed the SINV-P438L mutant also shown in Figure 2A. The SINV-P438L mutant virus was generated by plasmid transfection and compared with the WT virus. By infecting HOS and U2OS cells, it was noticed that the P438L mutation increased viral susceptibility in both cell types, with stronger fluorescence expression than WT SINV as shown in Figure 2B. Furthermore, SINV-P438L outperformed SINV in both infection efficiency and cytotoxicity in HOS and U2OS cells (Figures 2C and 2D), inducing caspase-positive cells more than twice those observed with SINV. In addition, infection of both cells with 1 MOI of SINV and SINV-P438L followed by fixation of the cells with agar to restrict viral spread to neighboring cells, SINV-P438L demonstrated higher propagation efficiency, with differences from WT SINV detectable as early as 10 min post-infection (Figure 2E). These results suggest that the mutation altered the transcriptional or replicative dynamics of the virus, thereby affecting viral protein expression in osteosarcoma cells. As the results present in Figure 2F, the viral titers of SINV-P438L were increased in HOS, U2OS, and MG-63 at 48 hpi. In Saos-2 cells, however, no superiority of the mutant was observed, likely SINV already exhibited high infection efficiency in Saos-2 cells, so mutants obtained by directed evolution have little gain on such cells.

Subsequently, we compared the tumor-killing capabilities of WT SINV and SINV-P438L in a HOS-derived subcutaneous tumor model in nude mice. Tumor volumes were regularly monitored and recorded before and after virus administration. As shown in Figure 2G, the tumor in mice in the PBS group developed rapidly, with most reaching volumes greater than 1,000 mm^3^ around 17 days after cells inoculation, accompanied by ulceration of the tumor tissue. In contrast, SINV-P438L treatment markedly delayed tumor progression, particularly at later stages, when the advantages of the mutant became increasingly apparent (Figure 2H). Tumor tissues excised 24 days after cell injection are shown in Figure 2I, providing a visual comparison of the post-treatment effects. From the results of H&E staining in Figure 2J, the tumor tissues from untreated groups showed densely arranged and highly heterogeneous tumor cells, whereas the tumors from SINV groups showed marked necrosis, including disappearance of nucleolus lysis. In contrast, SINV-P438L-treated tumors exhibited extensive necrosis, with only partial outline of the tumor cells remaining. Together, these results suggest that the P438L mutations located on nsP2 enhances the tumor-killing effect of SINV against osteosarcoma.

### Transcriptome analysis of SINV-P438L and SINV in osteosarcoma

**Figure 3:**
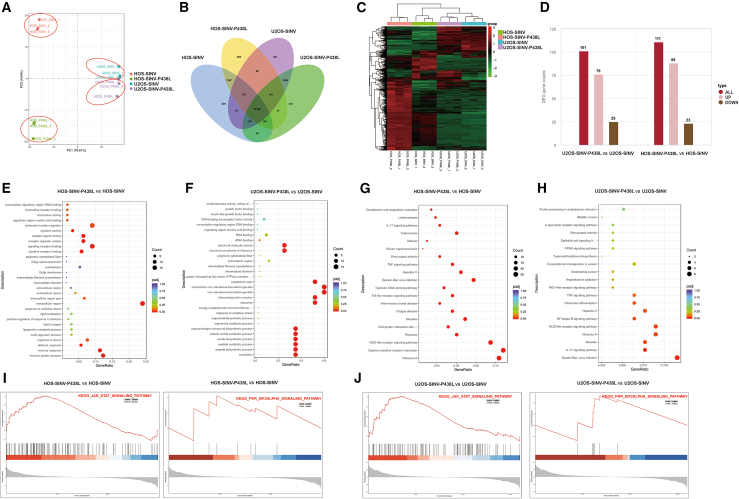
Effect of SINV-P438L infection on the cellular transcriptome (A) PCA of transcriptomes from HOS and U2OS cells infected with SINV or SINV-P438L (*n* = 3). (B) Venn diagram depicting the number of shared and unique DETs. (C) Heatmap of DEGs in SINV- and SINV-P438L-infected cells. The *x* axis indicates sample names, and the Y axis indicates normalized expression values of DEGs. Red represents higher expression, black represents medium expression, and green represents low expression. (D) Bar plot showing the number of identified DEGs. Pink represents upregulated genes and brown represents downregulated genes. (E and F) Gene Ontology (GO) enrichment analysis of DEGs. The *x* axis represents the ratio of the number of DEGs annotated to the GO pathway to the total number of DEGs; the *y* axis represents GO pathways. The size of the dots represents the number of genes annotated to the GO pathway and the *p*adj value ranges from 0 to 1, with lower values mean greater enrichment. (G and H) KEGG enrichment analysis of DEGs in HOS and U2OS cells after SINV and SINV-P438L infection. (I) GSEA showing enrichment of PKR-EIF2α and JAK-STAT signaling pathways in HOS cells following SINV or SINV-P438L infection. (J) GSEA showing enrichment of PKR-EIF2α and JAK-STAT signaling pathways in U2OS cells following SINV or SINV-P438L infection.

To investigate the mechanism of the increased efficiency of viral infection caused by the P438L mutation, HOS and U2OS were infected with SINV-P438L and SINV, and transcriptomes of the infected cell samples were sequenced. Three biological replicates were performed for each sample, and the PCA plots shown in Figure 3A illustrate the low variability within replicates of the same group. In addition, there was a marked divergence in transcriptomes between SINV-P438L-treated and SINV-treated HOS cells, whereas this difference was considerably smaller in U2OS cells. The sequencing results were further analyzed for differentially expressed genes (DEGs). As shown in Figure 3B, total of 10,163 genes were expressed in all four groups. SINV-P438L behaved similarly in HOS cells and U2OS, with approximately 500 genes differentially expressed compared with WT viruses. Figure 3C demonstrates the heatmap of hierarchical clustering of gene expression in the 4 groups. Differential expression analysis was conducted using DESeq2, identifying 111 upregulated genes, and 23 downregulated genes were screened in the HOS group under the criteria of |log2(FoldChange)| > 1 and *p* < 0.05. In the U2OS group, 76 upregulated genes and 25 downregulated genes were detected (Figure 3D). Gene Ontology analysis of DEGs revealed that, in the HOS cells, SINV-P438L significantly affected biological processes (BPs) associated with immune responses and stress responses, suggesting that the mutant viruses play a more significant role in activating cellular stress and immune response (Figure 3E). In addition, the P438L-induced alterations in cellular component (CC) and molecular function (MF) were mainly associated with cytokine activation, receptor activity, and extracellular region, suggesting that the mutation enhances virus-host interactions, thereby promoting immune evasion or augmenting immune activation by modulating receptor signaling pathways. In contrast, in U2OS cells, SINV-P438L was enriched in BPs such as translation, peptide metabolic process, and amide biosynthetic process, indicating that viral infection influenced host protein synthesis and peptide biosynthetic pathways (Figure 3F). The P438L mutation is differentially regulated in viruses during translation, potentially representing a viral adaptation strategy to enhance replication. Additionally, from the CC and MF results, the P438L mutation also affected RNA binding, ribosomes, and the ribonucleoprotein complex, suggesting that the mutated virus may promote replication by modulating transcription and translation processes in the host. Pathway enrichment analysis (KEGG) identified the top 20 significantly enriched pathways, as shown in Figures 3G and 3H. Most of the DEG-enriched pathways were associated with viral infection-related pathways, including Epstein-Barr virus, measles, and influenza A infections, as well as host interferon (IFN) signaling activated to mount antiviral responses.

Previous studies have shown that infection of host cells by SINV correlates with antiviral defense mechanisms, primarily the type I IFN-α/β and dsRNA-dependent protein kinase (PKR) pathways.^41^^,^^42^^,^^43^ Therefore, we focused on these two pathways for GSEA. In the PKR-EIF2α pathway, the highest enrichment was the EIF2AK2 gene, also known as the PKR gene. In the JAK-STAT pathway, enrichment was observed in both cell types for genes including IFNL1, IL23R, PIK3CG, SPRY4, and STAT1 (Figures 3I and 3J). Based on these results, we hypothesize that infection with the P438L mutant affects JAK-STAT signaling compared with WT SINV.

### The P438L mutation promotes the viral growth process by accelerating RNA synthesis

**Figure 4:**
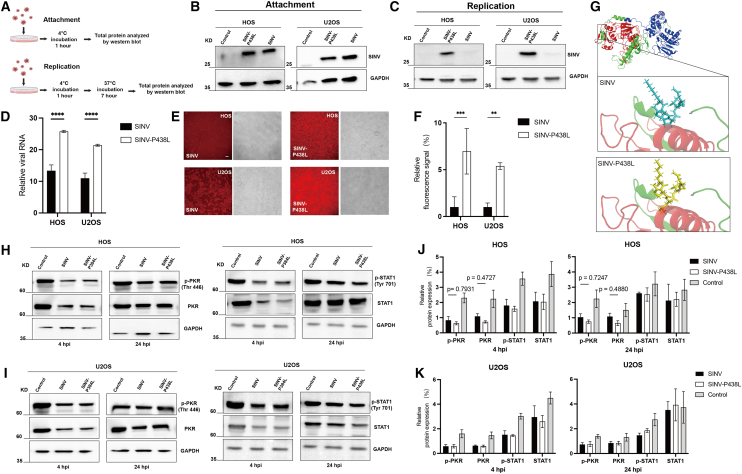
The P438L mutation in Nsp2 facilitated viral replication and transcription (A) Viral attachment and replication assays in HOS and U2OS cells (MOI = 1). (B and C). The results for attachment and replication assay, western blots were probed with a rabbit polyclonal anti-SINV serum generated by immunization of rabbits with purified SINV. (D) qPCR analysis of viral RNA replication (*n* = 4). (E) Representative images of nascent RNA synthesis in infected cells detected with RNA synthesis assay kit at 10 min post-infection (magnification, 10×). Scale bar, 100 μm. (F) Quantification of fluorescence intensity (*n* = 4). (G) AlphaFold2 structural prediction of nsP2. The highest pLDDT values of the predicted models were 91.7. The Pro-438 is labeled in stick representation. The 5 Å of the surrounding structure of wild-type SINV (cyan) and mutant P438L (yellow) is highlighted. (H and I) Western blot analysis of phosphorylated PKR, total PKR, phosphorylated STAT1, and total STAT1 in infected cells at MOI of 1 for 4 or 24 h. (J and K) Quantification of phosphorylated PKR, PKR, phosphorylated STAT1, and STAT1 expression.

The above results suggest that SINV-P438L may influence viral replication, transcription, protein synthesis, and host immune responses, compared with WT SINV. Therefore, the effects of the P438L mutation on virus attachment, replication, transcription, protein structure, and JAK-STAT pathways were further investigated. First, we collected cell samples infected with SINV and SINV-P438L at 4°C, exploiting the fact that viruses can only attach to the outer surface of host cells but cannot enter at low temperatures. Another set of samples was treated under the same conditions, after which viral supernatants were washed off thoroughly and cell were incubated at 37°C for an additional 7 h. Proteins were isolated for the analysis of viral replication (Figure 4A). As shown in Figures 4B and 4C, the P438L mutation did not affect viral attachment to the host cell but instead accelerated virus growth by enhancing intracellular replication. Quantification of viral RNA in cells also demonstrates that the mutant virus is altered at both the replication and transcriptional stages of early infection (Figure 4D). RNA synthesis was subsequently measured 10 min after WT and mutant viruses infected both HOS and U2OS cells (Figure 4E). Stronger RNA synthesis signals were observed in SINV-P438L-infected cells, and in HOS cells the fluorescence intensity of synthesized RNA was nearly 5-fold higher than that of WT SINV (Figure 4F). This suggests that the advantage of the mutant virus emerges at the early stage of infection, with the P438L substitution in nsP2 increasing replication and transcription efficiency, thereby enhancing cellular sensitivity.

Unlike other amino acids, the unique cyclic structure of proline contributes to the secondary structure of proteins. Substitution of Pro to Leu at position 438 is likely to alter nsP2 conformation. To evaluate this, we predicted the nsP2 structures of WT and SINV-P438L using AlphaFold 2. As shown in Figure 4G, the single 438 mutation is located at the end of a helical structure between the red-labeled deubiquitinase-like domain and the blue-labeled protease domain, and the root-mean-square deviation value of the two nsP2 protein structures is 1.035, which indicates that the overall three-dimensional conformation remained highly similar, with no significant structural change.

Previous results have shown that SINV infection is associated with host antiviral responses. Based on these findings, we focused on PKR antiviral pathway. Analysis of PKR phosphorylation and total PKR expression after infection revealed that SINV induced higher PKR phosphorylation levels than SINV-P438L at 4 hpi in HOS cells, and this upregulation persisted at 24 h (Figures 4H and 4J). However, these differences did not reach statistical significance. Since phosphorylated PKR can regulate the type I IFN pathway, we further examined STAT1, a key transcription factor in this pathway. No significant differences were detected in total STAT1 or p-STAT1 levels in either cell type following infection with WT or mutant virus (Figures 4I and 4K).

### Effects of SINV-P438L in other cell lines

**Figure 5:**
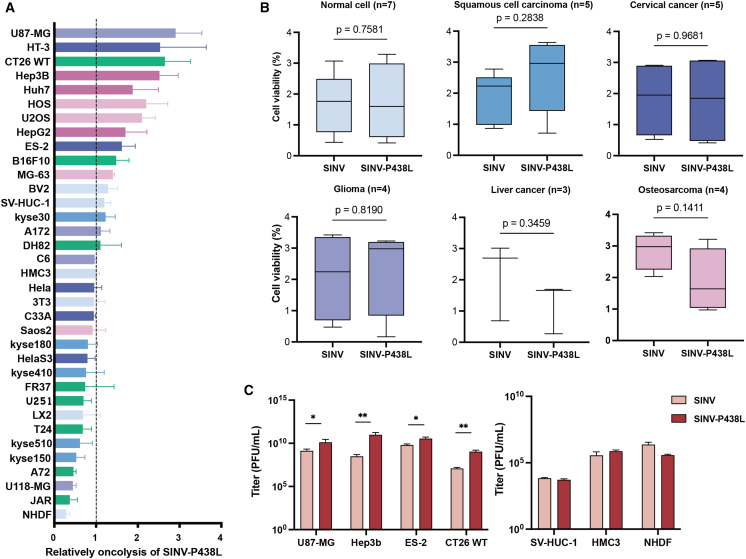
The tumor-killing ability of SINV-P438L was changed in variety of tumor cells (A) Cell viability of 35 tumor and normal cell lines after SINV or SINV-P438L infection (MOI = 0.1, 72 hpi). A ratio greater than 1 indicates enhanced tumor-killing ability of the mutant compared with the wild-type. Normal cells, light blue; squamous cell carcinoma cells, dark blue; glioma cells, purple; liver cancer cells, rose red; cervical cancer cells, cyan; osteosarcoma cells, pink. (B) Comparison of the oncolytic effects of SINV and SINV-P438L in various types of cell lines. Data were analyzed by a two-tailed unpaired *t* test, and results are presented as *p* values. The number of cell lines in each category was as follows: normal cells, *n* = 7; squamous cell carcinoma cells, *n* = 5; glioma cells, *n* = 4; liver cancer cells, *n* = 3; cervical cancer cells, *n* = 5; osteosarcoma cells, *n* = 4. (C) The growth pattern of SINV and SINV-P438L in U87-MG, ES-02, CT26 WT, Hep3b, SV-HUC-1, HMC3, and NHDF at 48 hpi (MOI = 0.01). ∗*p* < 0.05, ∗∗*p* < 0.01.

We next evaluated whether the oncolytic efficacy of SINV-P438L was limited to osteosarcoma cell lines or extended to other cell types. A total of 35 cell lines, including squamous cell carcinoma, glioma, liver cancer, cervical cancer, osteosarcoma, and normal cell, were tested for their response to SINV-P438L. At 72 hpi, the mutant virus exhibited greater cytotoxicity than WT SINV in 16 cell lines, including U87-MG, HT-3, CT26 WT, and Huh7 cells (Figure 5A). In contrast, the P438L mutation had no detectable effect on viral efficacy in 6 cell lines. Unexpectedly, SINV-P438L displayed reduced sensitivity in 13 cell lines, including NHDF, JAR, and kyse150. This suggests that the P438L substitution in nsP2 changes the sensitivity of SINV across diverse cell types.

To better visualize these effects, we clustered those cell lines and compare them in Figure 5B. The results revealed that SINV-P438L demonstrated improved tumor-killing efficiency in osteosarcoma and hepatocellular carcinoma cells. However, in squamous cell carcinoma cell lines, the oncolytic activity of SINV-P438L was slightly decreased. In addition, the P438L mutation did not significantly affect the cell killing effect of the virus in normal cell lines, glioma cell lines, and cervical cancer cell lines. Thus, SINV-P438L failed to broadly improve cytolysis in all tumor types, but the data provide an insight for selecting suitable tumor contexts for therapeutic application.

From these 35 cells, we further selected 7 representative lines, including 4 tumor-derived lines and 3 normal cells, to assess viral replication. These cells were infected with SINV and SINV-P438L, and the growth of the viruses was measured using plaque assay. As shown in Figure 5C, SINV-P438L replicated more efficiently than SINV in U87-MG, ES-2, CT26 WT, and Hep3B cells at 48 hpi, whereas no differences were observed in normal cells. These results suggest that SINV-P438L does not increase viral replication in normal cells, thereby reducing the risk of off-target cytotoxicity. In order to test the difference in the oncolysis capabilities of SINV and SINV-P438L *in vivo*, selected tumor cell lines will be used to establish mouse xenograft models for comparison with *in vitro* results.

### SINV-P438L has enhanced tumor-killing ability *in vivo*

**Figure 6:**
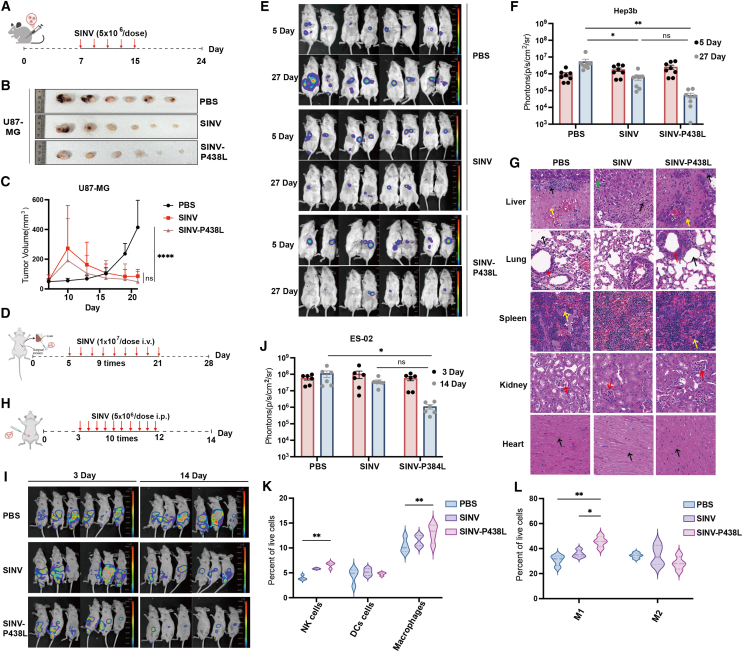
SINV-P438L improves tumor killing by different modes of delivery (A) Tumor growth in U87-MG xenografts treated with PBS, SINV, or SINV-P438L (*n* = 7). (B and C) Tumor length and width were recorded and volumes were calculated. Tumor tissues were collected at 24 days after cells transplantation. ∗∗∗∗*p* < 0.0001; ns, not significant. (D) Schematic representation of the liver cancer model construction and the schedule of drug administration (*n* = 8). (E) Representative IVIS images of Hep-3b-luc tumor-bearing mice at days 5 and 27. (F) Quantitative analysis of total whole-body photon counts in PBS-treated and SINV-treated groups. ∗*p* < 0.05, ∗∗*p* < 0.01. (G) On day 28 after cell injection, hearts, livers, spleens, lungs, and kidneys were collected from mice for H&E staining. In the liver, yellow arrows denote hepatocyte nuclear pyknosis and dissolution, black arrows indicate inflammatory infiltration, and green arrows show mild hepatocyte edema. In the lung, red arrows indicate bronchi, and black arrows indicate alveoli. In the spleen, yellow arrows highlight multinucleated giant cell infiltration. In the kidney, red arrows indicate glomeruli. In the heart, black arrows denote myocardial cells. Apart from the liver, the histological architecture of other major organs remained largely intact. Scale bar, 50 μm. (H) Schematic of ES2-Luc peritoneal metastasis model (*n* = 6). (I) Bioluminescence signal of ES2-Luc tumor-bearing mice before and after treatment. (J) Quantitative analysis of luciferase signal intensity corresponding to (I). (K and L) Flow cytometry analysis of NK cells, dendritic cells, and macrophage subpopulations in spleens of treated mice. DCs were labeled as CD11c- and MHC class II-positive cells. Macrophages were labeled by CD11b and F4/80 antibodies, and subsequently classified into CD86-positive (M1 macrophages) and CD206-positive (M2 macrophages) subpopulations (*n* = 4).

To demonstrate the therapeutic efficacy of SINV-P438L in tumor models beyond osteosarcoma, we established a U87-MG subcutaneous tumor model and performed the treatment as the process shown in Figure 6A. SINV-P438L suppressed tumor growth as shown in Figures 6B and 6C and compared with SINV, the mutated virus did not confer significant additional benefits. In addition, Hep3b-luc orthotopic liver tumor models were constructed and treated by tail vein injection according to the schedule shown in Figure 6D. Analysis of luciferase expression in tumor cells before and after treatment demonstrated that both viruses limited liver tumor progression, with SINV-P438L showing a trend toward stronger inhibition compared with WT SINV (Figures 6E and 6F). However, the differences did not reach statistical significance. In addition, autopsy results indicated that some Hep3B-inoculated mice developed peritoneal metastases, likely caused by minor cell leakage during transplantation. The results of H&E staining of the heart, spleen, kidney, and lungs revealed that repeated intravenous injections of SINV and SINV-P438L did not cause detectable pathological damage in these organs (Figure 6G). However, localized abnormalities were observed in the liver tissue at the tumor inoculation site, including hepatocellular necrosis and tumor infiltration. Subsequently, an ES-2 ovarian cancer peritoneal metastasis model was constructed and treated by intraperitoneal injection of PBS, SINV, and SINV-P438L for a total of 10 administrations (Figure 6H). The luciferase expression results demonstrate that SINV-P438L exhibited promising antitumor activity, with a trend toward lower photon counts compared with SINV, although the difference was not statistically significant (Figures 6I and 6J). To further examine immune modulation, spleens were collected from treated ES-2 tumor-bearing mice and lymphocytes were analyzed by flow cytometry. A significant increase in natural killer (NK) cells was observed in virus-treated mice, with a more pronounced enhancement in the SINV-P438L group (Figure 6K). This suggests that viral infection induces the secretion of IFN, thereby activating NK cells and coordinating innate immune responses to clear infected cells. In contrast, the number of dendritic cells (DCs) was not affected after treatment, and MHC class II expression analysis indicated that DCs did not activate antigen-presenting capacity. Consistent with previous studies,^44^ this likely reflects viral immune evasion through suppression of excessive DC activation, thereby maintaining viral persistence at a controlled level. In addition, analysis of macrophage polarization revealed distinct effects on M1 and M2 subsets. Treatment significantly increased M1 macrophages (pro-inflammatory response) (Figure 6L), whereas M2 macrophage (anti-inflammatory response) showed a slight decrease. This shift toward M1 polarization may reflect inflammatory activation induced by viral infection, thereby contributing to antitumor immunity and limiting tumor progression.

## Discussion

In this study, through directed viral evolution in both HOS and U2O cells, we generated an oncolytic SNV that more efficiently killed osteosarcoma cells *in vitro* and suppressed tumor growth in a diverse range of mouse models. In addition, we demonstrated that the P438L mutation in nsP2 enhances viral expression by promoting replication and accelerating RNA synthesis.

In the development of OVs, insensitivity in certain cancer cell lines limits their therapeutic scope. Directed evolution, a common method in virology research, is an efficient approach for obtaining the desired phenotypes. Through this process, viruses acquire adaptive mutations and evolve to become more suitable for growth in refractory tumor cells, thereby enabling more precise treatment of refractory tumors. This strategy has been applied in several studies to improve viral replication efficiency in specific tumor cells.^45^ For example, a mutant M1 virus with an approximately 6,000-fold increased infection efficiency was obtained after 10 serial passages of in HCT-116 cells.^46^ Serial passaging in human PDAC cell lines improved vesicular stomatitis virus (VSV) attachment and replication.^47^ Most of these studies have focused on RNA viruses due to their intrinsic characteristics of high mutation rates, rapid replication, and strong adaptability. These studies present the possibility to broaden the application scope of OVs.

Although a P438L substitution in nsP2 has not previously been described in engineered SINV, this mutation is known to occur in Ockelbo virus, a human-pathogenic strain of SNV. In SINV, nsP2 is an essential component of the replication complex, containing both deubiquitinase and protease activities.^42^^,^^48^^,^^49^^,^^50^ These enzymatic functions are required for polyprotein processing and for regulating negative- and positive-strand RNA synthesis during alphavirus replication. Most reported SINV nsP2 mutations map to structural region of the protein.^51^^,^^52^ For example, the P726G mutation reduced cellular pathogenicity and is associated with loss of nuclear function of nsP2 as well as reduced RNA synthesis and viral replication.^53^ In our study, the single substitution in nsP2 was likewise shown to alter viral replication and transcription efficiency in a cell-type-dependent manner. This finding suggests that nsP2 may represent a promising target for directed evolution of alphaviruses, both to improve tumor selectivity and for vaccine design. Although the precise mechanism by which the P438L enhances SINV remains unclear, its position between the deubiquitinase and protease domains suggests that this linker region may influence secondary structure folding, thereby affecting viral gene expression.

Our data indicate that both mutant and WT SINV exploit PKR inhibition to achieve efficient replication in osteosarcoma cells. PKR is a classical antiviral pathway activated by recognition of RNA intermediates generated during SINV replication.^41^^,^^44^ The observed phenotype reflects that both viruses can induce PKR phosphorylation, but the current data are insufficient to support the hypothesis that P438L enhances PKR activation. Considering the transcriptomic results, a more plausible interpretation is that the mutant influences replication dynamics and RNA synthesis rather than PKR signaling. In alphaviruses, nsP2 has also been implicated as a determinant of IFN-α/β release. Type I IFNs are major mediators of protection against alphavirus infection, rapidly restricting replication in DCs.^44^ STAT1 is a key transcription factor in this pathway, and our observation that STAT1 and p-STAT1 levels were maintained in HOS and U2OS cells supports that osteosarcoma cells are intrinsically resistant to SINV infection, which may contribute to their low permissiveness. Consistent with our data, we observed no significant differences in STAT1 or p-STAT1 between WT SINV and SINV-P438L. Prior work indicates that threonine 538 in nsP1, rather than residues in nsP2, is required for SINV-mediated suppression of STAT1 and inhibition of the JAK-STAT pathway, making a direct effect of P438L on this axis unlikely.^54^ We therefore infer that the mutant’s enhanced tropism arises primarily from modest increases in viral replication and altered IFN dynamics, rather than direct STAT1 antagonism. Moreover, osteosarcoma lines differ in IFN signaling states. Among them, U2OS and Saos-2 show impaired cGAS-STING-dependent induction,^55^^,^^56^ whereas HOS and MG-63 differ mainly in downstream ISRE and GAS responsiveness,^57^ which likely underlies their variable susceptibility to SINV and SINV-P438L.

Importantly, our panel of 35 cell lines revealed that SINV-P438L exhibits clear cell-type-specific tropism, with enhanced oncolysis in osteosarcoma and hepatocellular carcinoma, but reduced activity in squamous carcinoma and several other tumor types (Figure 5). This heterogeneity is unlikely to be explained solely by receptor expression. Instead, a previous study suggests that the permissiveness of tumor cells to SINV infection is largely determined by the integrity of antiviral pathways, particularly the type I IFN response.^54^ Based on this, we propose that the altered tumor specificity of SINV-P438L may result from changes in its ability to stimulate or evade IFN responses. Furthermore, even when viral replication is eventually curtailed by host immunity, SINV can elicit tumor-specific immune responses that contribute to durable antitumor activity.^54^ Thus, modulation of tumor-host IFN interactions by SINV-P438L may represent both a determinant of tumor tropism and a therapeutic advantage for precision virotherapy. Additionally, the cell-type-specific effects of SINV-P438L may involve unidentified host factors that interact with the deubiquitinase or protease domains of nsP2.^49^ Such cofactors could subtly influence viral polyprotein processing and replication efficiency, leading to differential susceptibility across tumor types.^48^

Interestingly, simultaneous passaging in two osteosarcoma cell lines obtained the same missense mutation, suggesting that P438L provides a strong adaptive advantage in the context of osteosarcoma. However, a limitation of the present study is that SINV-P438L were only evaluated in established cell lines and mouse models. Future work incorporating patient-derived samples will be critical to further assess the clinical relevance of the directed evolution strategy. Moreover, compared with other directed evolution studies such as in VSV that involved dozens of passages, our 10-passage selection may represent an early adaptive stage. Continued serial passaging in osteosarcoma or other resistant tumor types could accumulate additional beneficial mutations beyond P438L, further enhancing viral replication and tumor selectivity.

## Material and methods

### Cell

BHK-21 cells were obtained from the American Type Culture Collection. HOS, U2OS, Saos-2, MG-63, ES-2, and Hep3B cells were obtained from Procell Life Science & Technlogy (Wuhan, China). BHK-21, HOS, MG-63, and Hep3B cells were maintained in minimum essential medium (Thermo Fisher Scientific) with 10% fetal bovine serum (FBS) (Thermo Fisher Scientific) and 1% penicillin-streptomycin (Thermo Fisher Scientific). U2OS, Saos-2, and ES-2 cell lines were maintained in McCoy’s 5A medium with 10% FBS and 1% penicillin-streptomycin.

Hep3B-luc and ES-2-luc cell lines were constructed by lentiviral infection. The lentiviral vector encoding the firefly luciferase gene and EGFP gene was transfected into HEK293T cells to produce lentiviral particles. Afterward, Hep3b and ES-2 cell were sorted by flow cytometry to selected EGFP-positive cells, which were therefore luciferase-positive.

### Virus

The pSINV plasmids used in this study were identical to those described in our previous research.^26^ The mutation at amino acid position 438 on Nsp2 was inserted via two restriction enzyme sites, ClaI and SfiⅠ. pSINV and pSINV-P438L were transfected into BHK-21 cell using Lipofectamine 2000 reagent (Thermo Fisher Scientific), and the viral supernatants were collected when most BHK-21 cells exhibited marked CPEs at 72 h post-transfection, centrifuged at 2,000 × *g* for 5 min, and stored at −80°C.

Viral titer is measured using plaque assay. In brief, serially diluted viral suspensions were used to infect cells, and viral loads were quantified as plaque-forming units (PFU).

### Serial passaging of SNV

Totals of 1 × 10^6^ HOS and U2OS cells were inoculated into 6-well plates 1 day in advance. SINV was added at a MOI of 1, after which fluorescence expression and CPE were observed. Viral supernatants were collected at 72 hpi and plaque assay performed. In each passage, experiments were performed in biological triplicates. EGFP-positive and larger plaques were picked to infect HOS and U2OS cells, and viral supernatant harvested after 72 h was designated as P1. The procedure was repeated for subsequent passages (P2-P10). At each passage, plaque selection and reinfection were independently conducted in triplicate to ensure reproducibility. Sequencing of the P10 generation was performed after observing enhanced infection efficiency in HOS and U2OS cells.

### Cell viability assay

Tumor cells were inoculated in 96-well plates 1 day in advance to infection with SINV and SINV-P438L at MOI of 0.1 for 96 h. Ten microliters of MTT solution (5 mg/mL) was added to each well for 4 h according to the protocol of MTT cell proliferation and cytotoxicity assay kit (Beyotime Biotechnology). After that, 100 μL of formazan solution was added for 4–5 h until the formazan completely dissolved. Absorbance of the 96-well plate near 570 nm was measured using an enzyme-labeled instrument; lower absorbance values indicate greater cytotoxicity.

### Infection efficiency

Cells were infected with SINV or SINV-P438L at an MOI of 0.1, and cells were digested with 0.25% trypsin at 24 hpi, after which cell samples were washed three times with pre-cooled PBS. The proportion of cells in the cell suspension that were positive for EGFP at 488 nm was next analyzed using Flow Cytometers (Beckman, CytoFLEX).

### Animal models

The study was approved by the Animal Care and Use committees at Shenzhen Institute of Advanced Technology, Chinese Academy of Sciences, the Chinese Academy of Sciences (approval no. SIAT-IACUC-220809-NS-JF-A2177) in 2022. All mice were provided by Hunan SJA Laboratory Animal, Changsha, China (license no. SCXK [Xiang] 2019-0004).

Subcutaneous tumor model: 5 × 10^6^ HOS cells was injected subcutaneously into the right thigh of 4-week-old BALB/c nude mice. Seven days later, mice were randomly divided into different groups and injected with PBS, SINV (5 × 10^6^ PFU per dose), or SINV-P438L (5 × 10^6^ PFU per dose) three times, every other day. The volume of the tumor was measured regularly and calculated by the formula: tumor volume = 1/2 (length × width^2^).

Liver cancer model: NOD/SCID mice (male, 5–6 weeks) were anesthetized with 1% sodium pentobarbital. A 1–1.5-cm longitudinal incision was made below the xiphoid process, and 1 × 10^6^ Hep3B-luc cells were injected into the liver using a micropipette. Hemostasis was achieved by compression, and wounds were sutured, and the abdominal cavity was injected with 5 mL/kg gentamicin to prevent infection. After successful engraftment, mice were randomized into groups and treated via tail vein injection with PBS, SINV, or SINV-P438L (1 × 10^7^ PFU per dose) nine times. The growth of the tumor was monitored using the in vivo imager system (IVIS).

Peritoneal tumor model: 1 × 10^5^ ES-2-luc cells was injected intraperitoneally into 4-week-old BALB/c mice, the mice were then randomly divided into groups and treated with PBS, SINV (5 × 10^6^ PFU per dose), and SINV-P438L (5 × 10^6^ PFU per dose) via intraperitoneal injection, daily for 10 days. Tumor spread was monitored using IVIS imaging during the course of treatment.

For the *in vivo* administration of SINV, different injection routes were selected based on tumor models and clinical relevance to mimic the clinical administration of current oncolytic virus therapies. All animals were kept in a standard environment and euthanized when they lost more than 20% of body weight or when the tumor diameter exceeded 15 mm.

### IVIS imaging

For *in vivo* imaging, D-luciferin potassium salt (Yeasen, Shanghai, China) was administered at 50 mg/kg (luciferin/body weight) 10 min prior to the observation using the small animal IVIS. The results were analyzed using Living Image version 4.2.

### Flow cytometry analysis

Two days after SNV treatment in the ES-2 tumor-bearing mice, spleens were collected and placed in pre-cooled 1× HBSS (Gibco), after which 5 mL of mouse lymphocyte isolate (Dakewe, no. 711011) was added, and filtered through a 70-μm cell sieve (Corning) to make a cell suspension. Lymphocytes were isolated by density gradient centrifugation at 800 × *g* for 30 min, and the cells were counted after being washed with RPMI 1640 medium (Thermo Fisher Scientific). Afterward, a portion of the cell suspension was taken for fixation and cell membrane-breaking treatment, after which 1 μL of primary antibodies was added to the fractionated cell suspension and incubated on ice for 20 min in the dark. A Zombie Aqua Fixable Viability Kit (catalog no. 423101, BioLegend, San Diego, CA) was used for live/dead staining. Data were acquired using a Beckman CytoFLEX SRT and analyzed with FlowJo (v.10.8.1). The antibodies used in the experiments were as follows: FITC-anti-mouse CD4 (BioLegend, catalog no. 100510), PE anti-mouse CD49b (BioLegend, catalog no. 103506), FITC anti-mouse/human CD11b antibody (BioLegend, catalog no. 101205), PE anti-mouse F4/80 antibody (BioLegend, catalog no. 111603), brilliant violet 510 anti-mouse CD86 antibody (BioLegend, catalog no. 105039), APC anti-mouse CD206 (MMR) antibody (BioLegend, catalog no. 141707), and PE/cyanine7 anti-mouse CD11c antibody (BioLegend, catalog no. 117317).

### Western blot analysis

Cells were lysed in 1× SDS-PAGE loading buffer, after which samples were heated at 98°C for 5 min, and separated on 10% SDS-PAGE gels. The primary antibodies (1:2,500) were incubated at 4°C overnight after proteins had been transferred to a PVDF membrane. Then, HRP-conjugated secondary antibodies (1:5,000) were incubated for 2 h at room temperature. The antibodies used in the experiments were as follows: LAMR1/RPSA polyclonal antibody (Proteintech, no. 14533-1-AP), rabbit monoclonal anti-STAT1 (Cell Signaling Technology, no. 14994), rabbit monoclonal antiphospho-STAT1 (Cell Signaling Technology, no. 9167), rabbit monoclonal anti-PKR (Cell Signaling Technology, no. 12297), rabbit monoclonal antiphospho-PKR (Abcam, no. ab32036), HRP-conjugated goat anti-rabbit IgG (Proteintech, no. SA00001-2), HRP-conjugated goat anti-mouse IgG (Proteintech, no. SA00001-1), and anti-SINV primary antibody (laboratory generated). Purified SINV virions were used as immunogens to raise a rabbit polyclonal anti-SINV serum by intramuscular immunization with Freund’s adjuvant. Post-immune serum was collected and validated by western blot. For both immunofluorescence and western blot analyses, caspase-3 was detected using the same rabbit monoclonal antibody (caspase-3 antibody no. 9662, Cell Signaling Technology).

### Sequencing of transcriptome

SINV and SINV-P438L were used to infect HOS and U2OS cells at 1 MOI. RNA sequencing (RNA-seq) was performed at 12 hpi, when SINV replication had already initiated but widespread cell death had not yet occurred. This early-to-mid stage was chosen to capture viral RNA synthesis, replication complex activity, and host transcriptional responses while minimizing secondary effects caused by extensive cellular lesions. RNA was extracted from the collected cell samples and assessed using a NanoPhotometer spectrophotometer. RNA integrity was confirmed using an Agilent 2100 Bioanalyzer, and libraries were constructed with AMPure XP bead purification. After library construction, the quality of the library was assessed using a Qubit 2.0 Fluorometer and Agilent 2100 Bioanalyzer to ensure the library’s quality and successful sequencing, and the samples were pooled according to experimental needs and subjected to Illumina sequencing. Subsequently, the sequencing data were subjected to quality control and bioinformatics analysis.

### Attachment and replication assay

HOS and U2OS cells were infected with MOI of 1 of SINVs, after that, cells were incubated at 4°C for 1 h and cells were collected to analyzed the viral protein by western blot. For replication assay, cells were further incubated at 37°C for 7 h after initial 4°C incubation, and the proteins were tested by western blot.

### RNA synthesis assay

Following infection of HOS and U2OS cells with 1 MOI SINV and SINV-P438L, respectively, *de novo* RNA synthesis within cells was assessed using the BeyoClick EU RNA Synthesis Kit (containing AF647, Beyotime, catalog no. C10329S, China). Specifically, cells were incubated for 10 min with a 1 mM 5-ethynyluridine (EU) working solution. This uridine analog is incorporated into nascent RNA during transcription. Following incubation, cells were fixed with 4% paraformaldehyde for 15 min and then permeabilized with 0.5% Tween X-100 for 10 min. Incorporated EU residues were covalently conjugated to AF647-azide probes via a copper(I)-catalyzed click reaction (30 min, dark). Samples were then washed three times with the supplied washing buffer and imaged by fluorescence microscopy using excitation at 650 nm and emission at 670 nm.

### Protein structure prediction

Full-length SINV nsP2 amino acid sequences (WT and P438L variant) were modeled using AlphaFold2, deployed locally via Docker with the official reference databases (Uniref. 90, MGnify, BFD, PDB70). Predictions were run under the monomer_ptm preset with five independent seeds and three recycles. Template searches were performed against the PDB70 database without date restriction. Both unrelaxed and AMBER-relaxed models were generated using the built-in OpenMM protocol. The P438L substitution was introduced at the sequence level and modeled under identical conditions. Model confidence was evaluated using per-residue pLDDT, predicted aligned error, and predicted TM score. The top-ranked relaxed model based on mean pLDDT was selected and the top-ranked model was used for structural visualization. Structural visualization was performed in UCSF ChimeraX (v.1.x), with residue 438 highlighted and confidence scores mapped to surface color. These models were used only to illustrate the local mutation environment and to propose structural hypotheses, not as substitutes for experimental validation of proteolytic or enzymatic activity.

### Statistical analysis

All data are presented as mean ± SEM, and GraphPad Prism 8.0 was used for processing all graphs and statistical analysis of the data.

## Data and code availability

The data that support the findings of this study are available from the corresponding author upon reasonable request.

## Acknowledgments

We are grateful to Dr. Jean-Pierre Levraud (Macrophages and Development of Immunity, Institut Pasteur) for his kind gift of the SINV vector. This work was supported by the Shenzhen Key Laboratory of Viral Vectors for Biomedicine, Shenzhen Institute of Advanced Technology, 10.13039/501100002367Chinese Academy of Sciences (ZDSYS20200811142401005 to F.X.), the Guangdong Provincial Key Laboratory of Viral Biotechnology and Application (2022KSYS012 to F.X.), the 10.13039/501100021171Guangdong Basic and Applied Basic Research Foundation (2021A1515011235 to F.J.), the 10.13039/501100017607Shenzhen Fundamental Research Program (JCYJ20220818100801002 to F.J.), the Key Laboratory of Quality Control Technology for Virus-Based Therapeutics, Guangdong Provincial Medical Products Administration, Shenzhen (2020ZDB26 to F.X,), the Guangdong Provincial Medical Products Administration (2023ZDZ08 to F.X.), and the 10.13039/501100013289SIAT Innovation Program for Excellent Young Researchers (E1G023 to F.J.). Informed consent was not applicable to this study.

## Author contributions

F.J. and F.X. conceived of the project and supervised the project. K.S., Q.W., L.L., H.M., Z.R., and J.Y. preformed experiments, collected data, analyzed the data and discussion. K.S., and F.J. wrote the manuscript. All authors have read and approved the final manuscript.

## Declaration of interests

The authors declare no competing interests.
